# White Matter Deficits in Psychopathic Offenders and Correlation with Factor Structure

**DOI:** 10.1371/journal.pone.0072375

**Published:** 2013-08-20

**Authors:** Sylco S. Hoppenbrouwers, Arash Nazeri, Danilo R. de Jesus, Tania Stirpe, Daniel Felsky, Dennis J. L. G. Schutter, Zafiris J. Daskalakis, Aristotle N. Voineskos

**Affiliations:** 1 Department of Experimental Psychology, Utrecht University, Utrecht, Utrecht, The Netherlands; 2 Forensic Psychiatric Centre Oldenkotte, Rekken, Gelderland, The Netherlands; 3 Department of Psychiatry, Centre for Addiction and Mental Health, Toronto, Ontario, Canada; 4 Psychology Department, Correctional Services of Canada, Toronto, Ontario, Canada; Institute of Psychology, Chinese Academy of Sciences, China

## Abstract

Psychopathic offenders show a persistent pattern of emotional unresponsivity to the often horrendous crimes they perpetrate. Recent studies have related psychopathy to alterations in white matter. Therefore, diffusion tensor imaging followed by tract-based spatial statistics (TBSS) analysis in 11 psychopathic offenders matched to 11 healthy controls was completed. Fractional anisotropy was calculated within each voxel and comparisons were made between groups using a permutation test. Any clusters of white matter voxels different between groups were submitted to probabilistic tractography. Significant differences in fractional anisotropy were found between psychopathic offenders and healthy controls in three main white matter clusters. These three clusters represented two major networks: an amygdalo-prefrontal network, and a striato-thalamo-frontal network. The interpersonal/affective component of the PCL-R correlated with white matter deficits in the orbitofrontal cortex and frontal pole whereas the antisocial component correlated with deficits in the striato-thalamo-frontal network. In addition to replicating earlier work concerning disruption of an amygdala-prefrontal network, we show for the first time that white matter integrity in a striato-thalamo-frontal network is disrupted in psychopathic offenders. The novelty of our findings lies in the two dissociable white matter networks that map directly onto the two major factors of psychopathy.

## Introduction

In 1812, Benjamin Rush observed that individuals characterized by a psychopathy-like disorder were primarily afflicted by a ‘derangement in the moral faculties’ [1]. Recent conceptualisations of psychopathy suggest that it is a disorder characterized by callousness, egocentricity, grandiosity, often leading to profound antisocial behaviour [2]. Psychopathic offenders understand moral and societal rules, but show a striking indifference to those rules or any consequence of breaking them [3]. When confronted with moral choices, psychopaths show no reluctance to inflict personal harm if it helps to achieve their goals [4]. The extraordinarily destructive effects and massive societal cost of antisocial and psychopathic behavior underscore the paramount importance of making progress in our understanding of this disorder.

Factor analysis of one of the predominant conceptualizations of psychopathy, Hare’s Psychopathy Checklist-revised second edition (PCL-R) [5], reveal at least two dimensions of this disorder. Items that map onto Factor 1 denote interpersonal/affective traits, simplified with the term ‘emotional detachment.’ Factor 2 items are behavioural symptoms such as impulsivity or criminality simplified with the term ‘antisocial lifestyle’. Although the neurobiology of psychopathy in general is not well understood, recent data has begun to offer clues to the underlying circuitry disrupted in this disorder. Limbic structures such as the amygdala are strongly involved in emotion, the processing of emotionally laden information and the learning of conventional and moral rules [6]. Recent work [7] shows that psychopathic offenders have smaller amygdala volumes bilaterally, including deformations in the basolateral, lateral, cortical and central nuclei. By contrast, volumetric increases of the amygdala have also been observed in psychopathy [8]. The amygdala is connected with cortical regions that control and modulate subcortical functioning [9]. Connectivity between the amygdala and orbitofrontal cortex (OFC), and ventromedial PFC (vmPFC), is altered in psychopathic offenders and individuals with antisocial personality disorder [10]–[12]. The amygdala is connected to the prefrontal cortex by a white matter tract called the uncinate fasciculus, which is disrupted in psychopathic offenders [10]–[12]. In line, functional connectivity between the amygdala and the vmPFC is weaker in psychopathy [12] and in boys with psychopathic traits [13]. Furthermore, lesion studies show that damage to the vmPFC decreases the likelihood that individuals will avoid emotionally aversive outcomes [14]. In addition, during fear conditioning psychopaths show no significant activation of the amygdala-OFC network that is recruited in healthy controls [15]. (However, also see [16], [17]). Together these data imply amygdala-vmPFC dysfunction to partly underlie psychopathic behavior.

While evidence for disruption in amygdalo-prefrontal circuitry is mounting in psychopathy, others have shown alterations in temporal, parietal and limbic structures [18]–[20]. Recent functional neuroimaging work has implicated mesolimbic dopaminergic abnormalities in healthy individuals with psychopathic traits [21]. Healthy individuals with greater psychopathic tendencies have greater activation of the ventral striatum and anterior cingulate cortex during reward anticipation [22]. Impairments in the mesolimbic reward system were recently also found in violent offenders, suggestive of strong reward drives without consideration of potential punishment [23]. In addition, smaller volumes of the nucleus accumbens have recently also been observed in offenders with psychopathy [24]. Although disruption of the mesolimbic reward system has been associated with impulsive and violent behavior, this involvement has never been directly shown in psychopathic offenders, and certainly not in white matter circuitry.

The major objective of our study was to examine diffusion based measures of white matter throughout the brain in psychopathy to clarify the disrupted neural circuitry in this disorder. We completed diffusion tensor imaging (DTI) in psychopathic offenders and healthy controls, using a tract based spatial statistics (TBSS) approach that provides us with analysis of diffusion-based measures in white matter that is an optimized voxel-wise approach voxels [25] and is not limited by *a priori* assumptions regarding which circuitry is vulnerable in this disorder. Based on recent findings, we hypothesized that (i) psychopathic offenders would be characterized by disruption of white matter connecting amygdala to prefrontal cortex, and (ii) we would find disruption of white matter from nucleus accumbens to prefrontal cortex, corresponding to vulnerability of this network from recent functional imaging studies.

## Methods

### Participants

Psychopathic offenders (mean age ± standard deviation (SD); 33.5±7.4) were recruited from halfway houses in the Greater Toronto Area and through the Law and Mental Health Program at the Centre for Addiction and Mental Health (CAMH). Thirty-eight male offenders were interviewed of which 23 offenders met PCL-R inclusion criteria for this study. Offences included aggravated sexual assault, homicide, human trafficking and kidnap. Of these individuals, 12 psychopathic offenders reoffended within the duration of the study (October 20^th^ 2010–May 24^th^ 2011) and had either been arrested or were unlawfully at large. Therefore, 11 male psychopathic offenders were able to complete the entire study.

Psychopathic offenders were included if they scored 23 or higher on the Psychopathy Checklist-Revised edition (PCL-R) [5] (average ± SD; 28.1±3.3; range 23–34). In this community sample a relatively liberal cut-off of 23 was chosen to increase the number of subjects that could be enrolled in the study. This cut-off ensures moderate to strong psychopathic traits in criminal populations [5] and allows for a correlational approach to psychopathy which is beneficial with relatively small sample sizes. All included offenders have been administered PCL-R interviews conducted by trained, certified forensic psychologists and psychiatrists for clinical and risk assessment purposes. Exclusion criteria included schizophrenia or any primary psychotic disorder, bipolar disorder, depressive or anxiety disorders and other personality disorders, e.g., borderline personality disorder. Psychopathic offenders were also excluded if they currently met Diagnostic Statistical Manual-IV-TR (DSM-IV-TR) criteria for substance abuse disorder, that is, no history of substance abuse or dependence for at least 6 months. All offenders were subjected to regular drug screens as part of the terms of their parole which indicated none had abused drugs at the time during which they were participants in the study. During a standard safety screening [26] (done by SH) offenders were screened for the presence of neurological (e.g., traumatic brain injury, seizures, history of stroke), psychiatric disorders or contraindications to TMS, which were confirmed with a file review by an experienced clinician (done by TS). Right-handedness was confirmed with the Edinburgh Handedness Inventory [27]. All psychopaths had previously been administered the Shipley Institute of Living Scale and all scored at least in the average range (corresponding to a WAIS-R score ranging from 90–110 ([28]) indicating likely absence of organic brain damage. The Shipley Institute of Living Scale screens for organic brain damage and has a high correlation (r = 0.85) with the full scale WAIS-R [29].

Healthy age-matched male controls (mean age ± SD; 32.1±6) were recruited through advertisement in and around the Centre for Addiction and Mental Health (CAMH). Psychopathology including a lifetime history of substance dependence, or substance abuse in the past 6 months in the control group was also ruled out through a structured clinical interview for DSM-IV disorders (SCID-I) *(i.e. the* Structured Clinical Interview for DSM-IV Disorders [30]. All healthy controls also received a urine toxicology screen to rule out current substance use.

### Ethics Statement

In accordance with the Declaration of Helsinki the Research Ethics Board of CAMH approved of the study and written informed consent was obtained for all participants. The rights of all participants were protected. Psychopathic offenders were explicitly told that enrollment in or drop-out of the study would by no means interfere with their treatment or parole. All included psychopathic offenders received oral and written information about the study and were screened for contra-indications. If they wanted to participate they were asked to sign a Release of Information after which a review of previous psychological assessments was conducted to retrieve the PCL-R score. Hereafter, they were called to ask if they were still interested in participating: this usually gave psychopathic offenders a period of 2–3 days to reconsider their enrollment. Upon arrival to the lab, participants were again briefly told about the procedures and were then asked to sign the Informed Consent.

### Image Acquisition

DTI images were acquired using an eight-channel head coil on a 1.5 Tesla GE Echospeed system (General Electric Medical Systems, Milwaukee, WI), which permits maximum gradient amplitudes of 40 mT/m. A single shot spin echo planar sequence was used with diffusion gradients applied in 23 non-collinear directions and b = 1000 s/mm^2^. Two b = 0 images were obtained. Whole brain coverage was obtained (no gap), oblique to the axial plane. Slice thickness was 2.6 mm and voxels were isotropic. The field of view was 330 mm and the size of the acquisition matrix was 128 × 128 mm^2^, with echo time = 85.5 ms and repetition time = 15000 ms. To improve the signal to noise ratio, the entire sequence was repeated three times.

### Tract-based Spatial Statistics

The DTI data were converted to 4D NIfTI volumes. The resulting images for all three repetitions were merged for each subject, corrected for motion and eddy current distortion and ultimately averaged using standard tools available from FSL (www.fmrib.ox.ac.uk/fsl) [31]). After brain-extraction using BET [32], FA images were created by fitting a tensor model at each voxel to the averaged diffusion data using DTIFit (FMRIB’s Diffusion Toolbox), implemented in FSL. Voxelwise statistical analysis of the FA data was carried out using TBSS [25], part of FSL. All FA images were nonlinearly registered to the target image (FMRIB58_FA) provided by the FSL software. Next, the mean FA image was created and the tracts were narrowed to generate a mean FA skeleton which represents the centres of all tracts common to the all subjects. An FA threshold of 0.2 was chosen to discard non-white matter voxels [25]. The area surrounding the skeleton in each subject’s aligned FA map was searched perpendicular to the skeleton voxel and the locally highest FA values were projected onto the skeleton. This ensures that each subject’s skeleton remains in the group space while representing the centers of each individual’s own unique fiber tracts. The resulting individual skeletonised images were fed into voxelwise cross-subject statistics. Finally, group comparisons between subjects with psychopathy and normal controls were carried out with permutation-based analysis [33]. This was achieved with Randomise implemented in FSL, utilizing threshold-free cluster-enhancement method (TFCE) [34]. Statistical maps were then thresholded at P<.05 fully corrected for multiple comparisons (family-wiser error [FWE]-corrected). The most probable anatomical localizations for each cluster showing significant between-group differences in FA were determined with the FSL atlas tool using Talairach atlas, Harvard-Oxford cortical and subcortical structural atlases, and the JHU white matter tractography atlas (http://www.fmrib.ox.ac.uk/fsl/data/atlas-descriptions.html).

### Region of Interest Analysis

Region of interest (ROI) analyses were conducted as post hoc tests on mean FA values extracted from significant clusters resulting from TBSS. Independent-samples t-tests were performed on each cluster to assess group differences. In psychopathic offenders, partial correlation coefficients were then computed between mean FA values extracted from significant clusters (with more than 100 voxels) and PCL-R factors, while controlling for effects of age [35]. Statistical analyses were performed using SPSS v.17.0 for Windows (SPSS Inc., Chicago, IL).

### Probabilistic Tractography

To further elucidate the disrupted neural circuitry of psychopathy, significant different voxels between groups were used to seed the tractography algorithm. To generate individualized seed masks, significant voxels resulting from TBSS were projected back onto each control subject’s native space. The use of this approach (i.e. fiber tracking in healthy controls using abnormal voxel clusters identified in a disease group) has been recently described [36]. Probabilistic fiber tracking was conducted separately in each control subject using PROBTRACKX implemented in FMRIB’s Diffusion Toolbox (FDT). Using this algorithm, estimates of fiber orientation and their uncertainty were calculated at each voxel [37]. This model also accounts for the possibility of crossing fibers within each voxel (bedpostX) [38].

We used the default parameters with 5000 sample pathways per each seed voxel with a curvature threshold of 0.2 (corresponding to ±80°). Pathways were also terminated after 2000 steps, using a step length of 0.5 mm. Tractography results were then transformed to the Montreal Neurological Institute (MNI) space and averaged using the FA image that required the least warping among all other images during TBSS registration as the registration target [25]. Transformation fields from the TBSS registration stage were used to warp tractography results into the target image space. The transformed images were then averaged and linearly transformed to MNI standard space using the linear transformation matrix between the target image and the MNI standard brain. Finally, the resulting averaged image was thresholded to confine it to probabilities only greater than.5% of the maximum possible number of probabilistic pathways in a voxel (i.e. 5000 × total number of seed voxels).

## Results

**Figure 1:**
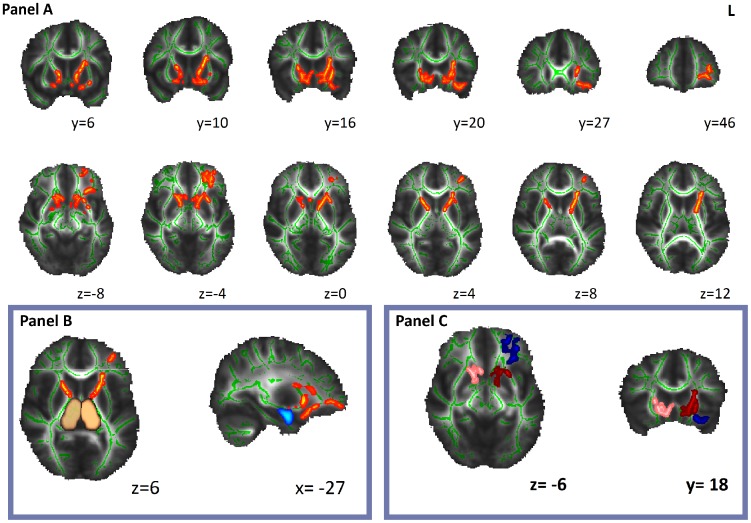
Panel A shows TBSS results with decreased anisotropy in psychopathic group as compared with normal controls (P_FWE_<0.05). **On an MNI brain (grayscale) the white matter skeleton is projected (green), as well as the location of significantly lower FA values in psychopaths as compared with normal controls (red-yellow).** Amygdala (light-blue) and thalamic nuclei (copper) derived from Harvard-Oxford subcortical atlas (panel B). At the bottom right Cluster 1 (dark-red), Cluster 2 (dark-blue) and Cluster 3 (pink) are shown (panel C).

The TBSS analysis showed five non-contiguous clusters of significantly decreased FA in white matter tracts of the psychopathic group as compared with controls (P_FWE_ <0.05) (Table 1, Figure 1). These clusters involved uncinate fasciculus and inferior occipitofrontal fasciculus bilaterally, extending towards both subgenual anterior cingulate (BA 25), left amygdala, left orbitofrontal cortex (BA47) and left frontal pole (BA10). In addition, decreased FA was also noted in regions involving bilateral anterior thalamic radiations and their medial extensions pointing to subgenual anterior cingulate. Cluster one corresponded to the subcortical and limbic portions of these white matter tracts (in amygdala, thalamus, subgenual anterior cingulate) and cluster two corresponded to the frontal components of these white matter tracts (in orbitofrontal cortex and frontal pole). The right sided cluster (cluster 3) stemmed from the anterior thalamic radiation and uncinate fasciculus with extensions to thalamus, striatum, pallidum and subgenual anterior cingulate. In general, FA deficits were more pronounced in the left hemisphere. Results showed no significant regions with increased FA value in psychopathic individuals compared with the controls.

**Table 1:** MNI atlas coordinates of clusters with significantly decreased FA values in the psychopathic group compared with the control group.

		MNI Coordinates (mm)					p values	
Clusters	Cluster size (mm^3^)	X	Y	Z	Associated WM Tracts	Associated Cortical and Subcortical Structures	Uncorrected	Corrected
**1**	1562	–15	12	–2	**Left ATR, IFOF, and UNF**	**Left thalamus, amygdala, striatum, and sACC**	1.6×10^–5^	8.2×10^–5^
**2**	887	–35	22	–16	**Left UNF, IFOF, and ATR**	**Left OFC and frontal pole**	1.8×10^–6^	9.0×10^–6^
**3**	793	15	16	–6	**Right ATR, UNF, and IFOF**	**Right thalamus, striatum, and sACC**	1.5×10^–6^	7.5×10^–6^
**4**	98	–33	45	5	**Left ATR**	**Frontal pole**	5.4×10^–4^	0.0027
**5**	14	–29	5	–9	**Left UNF**		0.0067	0.035

ATR = anterior thalamic radiation; UNF = uncinate fasciculus; IFOF = Inferior occipitofrontal fasciculus.

**Figure 2:**
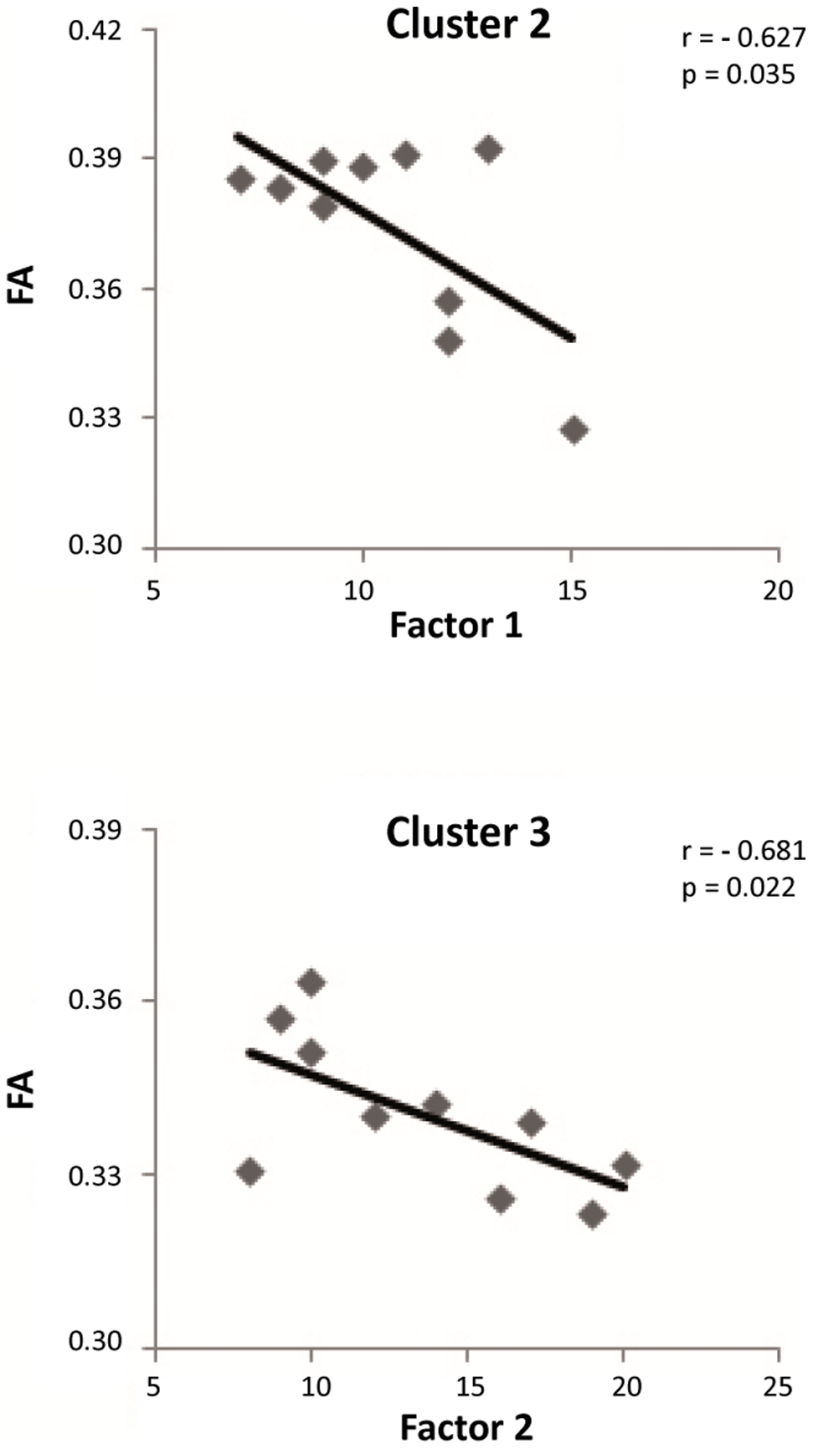
Scatterplots of the PCL-R subscores-mean FA correlations for Table 2 voxel clusters.

In the psychopathy group, we found significant correlations between PCL-R subfactors and mean FA values extracted from two of the clusters, while controlling for age (Table 2, Figure 2). Mean FA of the cluster 2, which mainly involved white matter in ventral regions of left frontal lobe, was negatively correlated with Factor 1 (r = –0.63, P_one-tailed_ = 0.04, n = 10). In addition, there was a negative correlation between mean FAs derived from the right-sided cluster (cluster 3) and Factor 2 (r = –0.68, P_one-tailed_ = 0.02, n = 10). However, there were no significant correlations between cluster 1 and PCL-R subscores. For one psychopathic offender only the overall psychopathy score was available. Therefore, correlational analyses were conducted in 10 psychopathic offenders.

**Table 2:** Partial correlation coefficients between mean FA values extracted from clusters that showed significant differences between psychopaths and healthy controls and one sided (p values) uncorrected for multiple comparison.

Mean FA values	PCL-R: total score	Factor 1	Factor 2
**Cluster 1**	0.353 (0.176)	–0.014 (0.485)	0.181 (0.321)
**Cluster 2**	–0.428 (0.125)	–0.627 (0.035)	–0.041 (0.458)
**Cluster 3**	–0.488 (0.091)	0.335 (0.189)	–0.681 (0.022)

**Figure 3:**
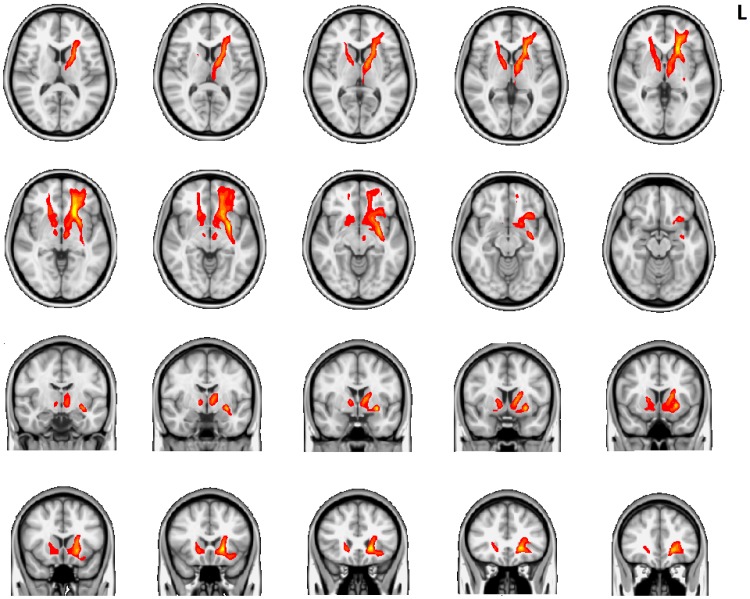
Probabilistic tractography results from healthy controls using seed voxels with abnormal FA in psychopathy. On a T1 MNI brain (grayscale) probabilistic tractography results (red-yellow) are demonstrated.

The probabilistic tractography seeded from voxels with abnormal FA encompassed the following pathways (Figure 3): (i) left and right ATR extending from thalamic nuclei rostrally towards frontal pole and medially to subgenual anterior cingulate and nucleus accumbens (ii) bilateral caudal continuation of the anterior thalamic radiation towards diencephalon and midbrain approaching medial ventral tegmental area/substantia nigra area (iii) left uncinate fasciculus bridging temporal pole (adjacent to the amygdala) and multiple regions in prefrontal cortex (including orbitofrontal cortex, frontal pole, ventromedial prefrontal cortex, and subgenual anterior cingulate cortex).

## Discussion

In this study, we show that psychopathic offenders have white matter deficits in two dissociable major networks. Namely, an amygdalo-prefrontal network (i.e., white matter tract connecting amygdala to prefrontal cortex) and a striato-thalamo-frontal network (a network connecting the nucleus accumbens, thalamus and prefrontal cortex). Importantly, correlations between the underlying factor structure of psychopathy and white matter tracts were found: we found correlations between (i) PCL-R Factor 1 and integrity of projections from amygdala to medial prefrontal and orbitofrontal cortex, and (ii) between PCL-R Factor 2 and projections from ventral tegmental area and nucleus accumbens through the anterior limb of internal capsule to thalamus and prefrontal cortex.

Earlier structural and functional MRI studies have repeatedly implicated disruption of an amygdalo-prefrontal network in psychopathy. Indeed, our findings align with recent data from antisocial personality disorder [11], children with callous-unemotional traits [39], boys with conduct disorder [40] and psychopathic offenders [10], all pointing to disrupted white matter within these regions. Recently, disruption of the uncinate fasciculus, which connects amygdala to vmPFC and OFC was implicated in psychopaths, supporting our findings [10], providing evidence for the theory that the social and emotional deficits in psychopaths reflect a deficient interaction between OFC/vmPFC and amygdala [6]. A subsequent study that included the same 9 psychopathic individuals from the Craig et al study, plus another six (total n = 15) with antisocial personality disorder found white matter disruption in frontally-based white matter tracts [11] connecting to the limbic system, providing further support for this notion. Furthermore, a recent study of boys with psychopathic tendencies found reduced white matter concentration in frontal cortex [41], raising the possibility that even mild neural alterations may lead to subclinical behaviors along a continuum.

While evidence continues to accumulate for disruption in prefrontal structures and connections from prefrontal cortex to amygdala, there is substantial evidence for structural and functional disruption of the amygdala itself in psychopathy and related disorders. In youths with both conduct disorder and strong psychopathic traits, the amygdala and the OFC are shown to be significantly less active during early reinforcement learning [13]. Blair has suggested that in psychopaths a dysfunctioning amygdala is pivotally important as it results in poor fear conditioning and passive avoidance learning [42], both of which are implicated in the pathogenesis of psychopathy [6]. In fact, poor fear conditioning in childhood is significantly predictive of adult criminal behaviour suggesting a neurodevelopmental aspect of amygdala damage in psychopathy [43]. Interestingly, a recent study suggests that functional connectivity deficits in limbic areas may already be present at early age and may prelude structural connectivity deficits [44]. Within the limbic system, significant bilateral volume reductions in the amygdala, accompanied by surface deformations in specific amygdalar nuclei provide further evidence for disruption of this core circuitry which correlate strongly with the affective and interpersonal aspects of psychopathy [7]. When our findings are taken together with the existing literature, it is becoming increasingly clear that structural disruption of white matter in the OFC, vmPFC and frontal pole, and white matter connections to these regions from the limbic system may explain the dysfunctional interpersonal and affective aspects of psychopathy.

Although some parts of the mesolimbic reward system (e.g., the striatum) have been implicated in psychopathy, our implication of disrupted white matter in a striato-thalamo-frontal network is new. Therefore, the damage in white matter tracts connecting the nucleus accumbens and ventral tegmental area through the anterior limb of the internal capsule to the thalamus and frontal cortex highlights a key second system that may be disrupted in psychopathic offenders. Affected white matter fibers extending to the midbrain appear to parallel those of the medial forebrain bundle, which connect ventral tegmental area with the nucleus accumbens - an important part of reward circuitry [45]. Indeed, we found that disruption in striato-thalamo-frontal circuitry was correlated with Factor 2 of the PCL-R. Other structural MRI studies have found increased volume of the striatum [46], albeit in different parts [23], [47], [48]. Recent accounts of striatal functioning in antisocial populations have suggested a particular pattern of impairment [49]. While normal controls recruit activity in the anterior cingulate when stimuli lose their rewarding properties, antisocial individuals retain activity in the striatum [50]. Once these individuals are motivated to obtain a certain reward, striatal impairments may result in the inability to flexibly use contextual information to terminate certain behaviors, for instance aggressive or antisocial behavior [49]. A recent study examined mesolimbic dopamine reward system functioning in healthy individuals with psychopathic traits [21]. Using a combination of PET and fMRI in the same individuals, neurochemical and neurophysiological hyperreactivity of the dopaminergic reward system was correlated with impulsive-antisocial temperament, providing potential explanations for increased substance use, impulsivity (however, also see [51]), and violence in this population [21]. Moreover, dopaminergic networks in the VTA and striatum correlate with trait impulsivity, where higher dopamine release in the striatum is predictive of stronger individual desire for drugs [52]. By contrast, Kiehl and colleagues [53] found reduced functioning of the striatum in psychopathic offenders but, as stipulated by Glenn and Yang [49], this may have to do with the negatively valenced stimuli that were presented. It is important to note that it is unclear whether hyperreactivity of the mesolimbic reward system in healthy individuals would induce the same behavior as white matter disruption of this circuit in psychopathic offenders. That is, white matter damage in a certain network is not typically associated with hyperreactivity. Nonetheless, when our data are taken together with these recent findings, evidence suggests that disruption of the reward system circuitry may underlie a group of behaviors common to psychopathic offenders, including aggression/violence and impulsivity/substance abuse.

An important limitation of our study was the relatively small sample size for a neuroimaging study. Although 23 individuals had consented to our protocol, nearly half re-offended during the relatively brief time course of the study. However, this is consistent with the nature of this population, and could be considered a reflection of the severity of psychopathic and antisocial behavior in our sample. Another important limitation is that we did not control for a past history of substance abuse in comparing psychopathic offenders to controls, and there is good evidence to suggest that effects of substances themselves can influence white matter measures [23]. A recent study, however, shows that gray matter deficiencies in mesolimbic circuitry in psychopaths are associated with violent behaviour and psychopathy and may be relatively independent of substance abuse whereas integrity of prefrontal regions such as the OFC may be more susceptible to drug use [23]. The drive for substance abuse itself may therefore be a component of the disorder of psychopathy, and is not easy to separate. Importantly, psychopathic offenders who completed all protocols were not using substances during the time period of the study, as they were subject to regular drug screens as a condition of their parole. One approach for future studies would be to include a third group of non-psychopathic offenders with a history of substance use as comparators. Finally, our main dependent measure of fractional anisotropy does not reveal the cellular correlates within white matter that are disrupted, as it can only indirectly index microstructural integrity of white matter. Changes in FA could be due to alteration of axonal membranes, changed axon number, disruption of the myelin sheath, or all of the above. Post-mortem investigations in this population combined with post-mortem DTI scanning could be especially valuable in elucidating these correlates, which may then be translatable to potential treatment.

In summary, we found profound disruption of white matter circuitry in psychopathic offenders compared to healthy controls. Our data suggest that the neural correlates of the interpersonal affective dysfunction lies in disruption of amygdalo-prefrontal circuitry and the neural correlates of antisocial/violent behavior lie in disruption of striato-thalamo-frontal reward circuitry that is tightly linked to impulsivity and substance abuse. The extent of white matter damage attributed to neurodevelopment versus that acquired over time in psychopathy is a potentially exciting area of future investigation that deserves further exploration.
